# Secondary Infections in Cancer Patients with Febrile Neutropenia

**DOI:** 10.5152/tjh.2011.75

**Published:** 2012-10-05

**Authors:** Alpay Azap, Gülden Yılmaz Bozkurt, Meltem Kurt Yüksel, Hakan Kutlu, Pervin Topçuoğlu, Adalet Aypak, Hamdi Akan

**Affiliations:** 1 Ankara University, School of Medicine, Department of Infectious Diseases and Clinical Microbiology, Ankara, Turkey; 2 Medicana International Ankara Hospital, Ankara, Turkey; 3 Ankara University, School of Medicine, Department of Hematology, Ankara, Turkey; 4 Ankara Numune Education and Research Hospital, Department of First Infectious Diseases and Clinical Microbiology, Ankara Turkey

**Keywords:** Hematologic malignancy, Febrile neutropenia, Secondary infection

## Abstract

**Objective:** Patients with neutropenia due to cancer chemotherapy are prone to severe infections. Cancer patients canexperience >1 infectious episode during the same period of neutropenia. This study aimed to determine the etiologicaland clinical characteristics of secondary infectious episodes in cancer patients with febrile neutropenia and to identifythe factors associated with the risk of secondary infectious episodes.

**Material and Methods:** All cancer patients that received antineoplastic chemotherapy at Ankara University, School ofMedicine, Department of Hematology between May 2004 and May 2005 and developed neutropenia were included in thestudy. Data were collected using survey forms that were completed during routine infectious diseases consultation visits.Categorical data were analyzed using the chi-square test, whereas Student’s t-test was used for continuous variables.Multivariate logistic regression analysis was performed to identify independent predictors of secondary infections (SIs).

**Results:** SIs were observed during 138 (53%) of 259 febrile neutropenic episodes. Of the 138 episodes, 89 (64.5%)occurred in male patients with a mean age of 40.9 years (range: 17-76 years). In total, 80% of the SIs were clinically ormicrobiologically documented. Factors on d 4 of the initial febrile episode were analyzed via a logistic regression model. The presence of a central intravenous catheter (OR: 3.01; *P<0.001*), acute myeloid leukemia (AML) as the underlyingdisease (OR: 2.12; *P=0.008*), diarrhea (OR: 4.59; *P=0.005*), and invasive aspergillosis (IA) during the initial febrileepisode (OR: 3.96; *P=0.009*) were statistically significant risk factors for SIs.

**Conclusion:** Among the cancer patients with neutropenia in the present study, AML as the underlying disease, thepresence of a central venous catheter, diarrhea, and IA during the initial febrile episode were risk factors for thedevelopment of SIs.

## Abstract (tr)

**Amaç:** Antineoplastik kemoterapi uygulanan kanser hastalarının hemen tamamında nötropeni gelişmektedir. Nötropenik dönemde birden fazla febril atak ortaya çıkabilmektedir. Bu çalışmada, nötropenik hastalarda ilk febril atak sonrasında gelişen ikincil atakların etiyolojik ve klinik özellikleri ile birlikte sekonder infeksiyon için risk faktörlerinin belirlenmesi amaçlanmıştır.

**Gereç ve Yöntemler:** Ankara Üniversitesi Tıp Fakültesi Hematoloji AD’da çeşitli maligniteler nedeniyle 2004 Mayıs ve 2005 Mayıs tarihleri arasında kemoterapi uygulanan ve febril atak/ataklar gelişen hastalar çalışmaya dahil edilmiş ve bu hastaların kayıtları geriye dönük olarak taranmıştır. Kategorik değişkenler ki-kare testi, devamlı değişkenler ise Student’s test uygulanarak değerlendirilmiştir. İkincil febril atak için risk faktörlerini belirlemede lojistik regresyon analizi uygulanmıştır.

**Bulgular:** Çalışma periyodunda gelişen 259 febril nötropenik atağın 138’inde (%53) sekonder infeksiyon gözlemlenmiştir. Sekonder infeksiyon gelişen hastaların %64.5’i erkek iken ortalama yaş 40,9’dur (17-76). Gelişen sekonder infeksiyonların yaklaşık %80’i klinik ya da mikrobiyolojik dokümante infeksiyonlardır. İlk ateş atağının 4. gününde incelenen faktörler için lojistik regresyon analizi uygulandığında; sekonder infeksiyon gelişimi için risk faktörleri olarak sanral venöz kateter bulunması (odds oranı [OR], 3.01; P < 0.001), altta yatan hastalık olarak Akut Myeloid Lösemi (AML) olması (OR, 2.12; P = 0.008), diare olması (OR, 4.59; P = 0.005) ve ilk atakta invazif aspergilloz (İA) tanısı bulunması (OR, 3.96; P = 0.009) belirlenmiştir.

**Sonuç:** Santral venöz kateter bulunması, altta yatan hastalığın AML olması, diare görülmesi ve ilk atakta İA gelişmiş olması nötropenik hastalarda sekonder infeksiyon riskini arttırmaktadır.

## INTRODUCTION

During the last 2 decades major developments in the treatment of malignant diseases have been realized, including bone marrow transplantation, aggressive antineoplastic treatment modalities, and the use of intravascular catheters. Nonetheless, all these developments are associated with longer periods of neutropenia, severe mucositis, and widespread use of antimicrobial therapy or prophylaxis, which probably lead to the emergence of drug-resistant microorganisms. Consequently, patients undergoing such treatment are prone to severe infections, and cancer patients can experience multiple infectious episodes during the same period of neutropenia [1,2].

Recently, consideration of the level of risk of severe infection in patients treated for febrile neutropenia has been strongly recommended. To date, several studies have sought to identify severe infection risk factors in febrile neutropenia patients, and mortality rates in febrile neutropenia patients have been reported; however, only a few studies examined the factors associated with the risk of secondary infections (SIs) [1,2,3,4,5].

As such, the aim of the present study was to determine the etiological and clinical characteristics of SIs in cancer patients with neutropenia, and to identify the factors associated with the risk of SIs.

## MATERIALS AND METHODS

All cancer patients that received antineoplastic chemotherapy at Ankara University, School of Medicine, Department of Hematology between May 2004 and May 2005 and developed neutropenia were included in the study. The local ethics committee approved the study. Fever was defined as ≥2 axillary temperature measurements of ≥38 °C within a 12-h period or 1 measurement of ≥38.5 °C. Patients were considered to be neutropenic if their neutrophil count was <500 cells mm^–3^, or <1000 cells mm^–3^ and expected to decrease to <500 cells mm^–3^ within 24-48 h. SI was defined as follows: i) any clinical or microbiologically documented infection that did not exist at the time of initial evaluation, but developed during empirical therapy or within 1 week after discontinuation of therapy, and ii) fever that responded to empirical therapy, but recurred after an afebrile period of 48 h during empirical therapy [1,6]. 

Each primary infection (PI) and SI was classified as a microbiologically documented infection (MDI), clinically documented infection (CDI) (objective and detectable signs of infection with a lack of microbiological documentation), or fever of unknown origin (FUO). Variables assessed on d 4 of the PI were age, sex, underlying hematological disease, comorbid diseases, receipt of a bone marrow transplant, severity of neutropenia, presence of a central venous catheter (CVC), history of fungal infection prior to the study, presence of oral mucositis, presence of diarrhea (>3 times d^–1^), presence of a catheter-related infection, administration of antibacterial, antiviral, and antifungal prophylaxis, antimicrobials used during the treatment of PIs, presence of invasive fungal infections, type of infection documentation (MDI, CDI, or FUO), and, if MDI, the infectious microorganism. Episodes of SI wereevaluated in terms of the type of infection documentationand the infectious microorganisms.

Data were collected using structured survey forms thatwere completed during routine infectious diseases consultationvisits. Categorical data were analyzed via the chisquaretest, whereas Student’s t-test was used for continuousvariables. Multivariate logistic regression analysis wasperformed to identify independent predictors of SIs. Datawere analyzed using Stata v.8.0 statistical software (StataCorp., Texas, USA) and the level of statistical significancewas set at P<0.05, using two-sided comparisons.

## RESULTS

SIs were observed during 138 (53%) of 259 febrile neutropenic episodes. Of the 138 episodes, 89 (64.5%) occurred in male patients with a mean age was 40.9 years (range: 17-76 years). Acute myeloid leukemia (AML) (64.5%) was the most common underlying disease, followed by acute leukemoid leukemia (16.5%), lymphoma (11.8%), and others (multiple myeloma, myelodysplastic syndrome, chronic myeloid leukemia, and aplastic anemia) (7.2%). In all, 14% of the patients had undergone bone marrow transplantation and only 1 patient had a comorbid disease (diabetes mellitus). 

Among the episodes of PI, 121 (47%) were FUO, 73 (28%) were MDI, and 65 (25%) were CDI. The microbial agents responsible for PIs were gram-positive bacteria (54%, n=35), gram-negative bacilli (43%, n=28), and fungi (3%, n=2). Possible and probable invasive aspergillosis (IA) was diagnosed in 35 (14%) of the patients, based on European Organization for Research and Treatment of Cancer (EORTC) criteria [7]. Among the episodes of SI, 81 (59%) of the 138 episodes were CDI, 27 (20%) were MDI, and 30 (21%) were FUO. In total, 27 bacterial pathogens were isolated, of which 12 (44%) were gram-positive bacteria and 15 (56%) were gram-negative bacilli. No fungi were isolated. 

The baseline characteristics of the febrile neutropenic patients and SI episode rate according to these characteristics are presented in Table 1. Univariate analysis was performed in order to identify the risk factors for SI and included such variables as gender, age, diarrhea, neutrophil count, grade 3-4 mucositis, IA during the PI, CVC, CDI, or MDI during the episode of PI, and AML as the underlying disease. Among these factors, univariate analysis showed that diarrhea (P<0.001), IA during the episode of PI (P<0.001), CVC (P<0.001), CDI or MDI during the episode of PI (P=0.009), and AML as the underlying disease (P=0.001) were strongly correlated with SIs. Neutropenia and severe neutropenia (<100 mm–^3^) were not associated with SIs. 

The baseline risk factors during PI were analyzed using a logistic regression model of multivariate analysis. The presence of a CVC (OR: 3.01; P<0.001), AML as the underlying disease (OR: 2.12; P=0.008), diarrhea (OR: 4.59; P=0.005), and IA during PI (OR: 3.96; P=0.009) were statistically significant risk factors for SIs (Table 2).

## DISCUSSION

Identification of the risk factors for infection is a logical approach in the treatment of febrile neutropenic patients. Although various studies have reported these risk factors, SIs were considered in only a few [1,2,3,4,5,8,9,10,11]. The frequency of SIs in adults varies between 12% and 24%. Recently Paganini et al. reported an SI rate of 17% in children [4]. In the present study the SI incidence rate was much higher—56%. This may have been related to underlying disease and the chemotherapeutic regimen, as AML was the underlying disease in 64.5% of the patients and 14% patients had undergone bone marrow transplantation. At the time of the study prophylactic use of such antibacterial agents as quinolones was not practiced at our institution, which may also be a factor related to the high incidence of SIs in the present study. 

AML as the underlying disease, presence of a CVC, diarrhea, and IA during PI were associated with the development of SIs in the present study. The presence of a CVC was previously reported to be a marker of SIs [1,2]. CVCs may promote skin and soft tissue infections, and are associated with bacteremia, especially due to gram-positive pathogens [12,13]. As empirical treatment regimens used at the onset of an episode of febrile neutropenia do not usually target gram-positive bacteria, CVC is a reliable risk factor for SI; however, Serra et al. reported that there wasn’t a relationship between the presence of CVC and the development of SIs [14].

The other independent factor associated SIs in the present study was AML as the underlying disease. Patients with AML as the underlying disease were administered more intensive chemotherapeutic regimens than the other patients in the study and therefore were more prone to SIs. The risk of SIs increases significantly in the presence of diarrhea; the integrity of the gastrointestinal mucosa is compromised and bacterial translocation occurs more easily in patients with diarrhea. Serra et al. reported that the duration and severity of neutropenia are risk factors for developing SIs [14]. The duration of severe neutropenia (<100 neutrophils mm^–3^) was strongly associated with SIs in a study by Nucci et al. [2]. Nevertheless, neutropenia (<500 neutrophils mm^–3^) and severe neutropenia were not associated with SIs in the present study. With respect to the present findings, Feld et al. reported that the duration of neutropenia had no effect on the development of SIs [15]. Akova et al. reported that the neutrophil count on d 0 was not a predictive factor for SIs, whereas persistently and severely neutropenic patients were prone to SIs [1].

IA during PI was also a risk factor for SIs in the present study. SIs occurred in 36 of the 49 patients with a history of IA. Such an association has not been previously reported, and might be due to the fact that IA usually occurs in high-risk patients with profound neutropenia for a long time (>10 days) [16]. On the other hand, the duration (>10 days) and severity (<100/mm^-3^) of neutropenia were not observed to be independent risk factors for SIs based on statistical analysis of the present study’s data. The effect of the duration and severity of neutropenia on SI might have been obscured by the high incidence of IA among the patients included in the present study.

The present study has some limitations. First, stage of the underlying disease and chemotherapeutic regimens (remission induction or consolidation) were not included in the statistical analysis. The stage of underlying disease and chemotherapeutic regimens have major impact on the duration and severity of neutropenia, both of which were not associated with SIs in the study. As such, the stage of underlying disease was not considered to be an important factor. Second, as the study group consisted of adults with a mean age of 40.3 years, only 1 patient had comorbid disease (diabetes mellitus). As such, it was not possible to assess the association between comorbidities and the development of SIs.

In conclusion, although SIs in neutropenic patients are common, they are rarely studied. The present study analyzed SIs in febrile neutropenic patients to identify the risk factors for SIs. AML as the underlying disease, presence of a CVC, diarrhea, and IA during PI are factors that may be considered as risk factors for SIs in neutropenic patients. 

**Conflict of Interest Statement **

The authors of this paper have no conflicts of interest, including specific financial interests, relationships, and/ or affiliations relevant to the subject matter or materials included.

## Figures and Tables

**Table 1:**
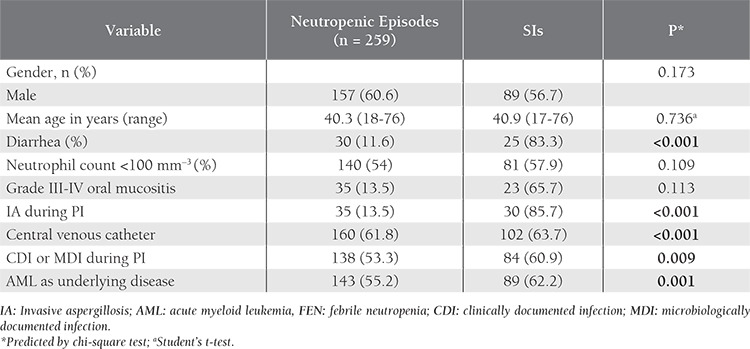
Characteristics of the febrile neutropenic patients and the SI rate according to these characteristics

**Table 2:**
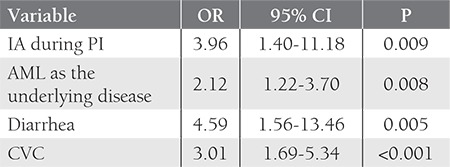
Multivariate analysis via logistic regression modeling of the factors associated with SIs

## References

[1] Akova M, Paesmans M, Calandra T, Viscoli C (2005). International Antimicrobial Therapy Group of the European Organization for Research and Treatment of Cancer: A European Organization for Research and Treatment of Cancer-International Antimicrobial Therapy Group Study of secondary infections in febrile neutropenic patients with cancer. Clin Infect Dis.

[2] Nucci M, Spector N, Bueno AP, Solza C, Perecmanis T, Bacha PC, Pulcheri W (1997). Risk factors and attributable mortality associated with superinfections in neutropenic patients with cancer. Clin Infect Dis.

[3] Freifeld AG, Bow EJ, Sepkowitz KA, Boeckh MJ, Ito JI, Mullen CA, Raad II, Rolston KV, Young JA, Wingard JR, Infectious Diseases Society of America (2011). Clinical practice guideline for the use of antimicrobial agents in neutropenic patients with cancer: 2010 update by the infectious diseases society of america. Clin Infect Dis.

[4] Paganini H, Caccavo J, Aguirre C, Gómez S, Zubizarreta P (2011). A scoring system to predict superinfections in high-risk febrile neutropenic children with cancer. Bol Med Hosp Infant Mex.

[5] Paesmans M (2000). Risk factors assessment in febrile neutropenia. Int J Antimicrob Agents.

[6] Hughes WT, Armstrong D, Bodey GP, Bow EJ, Brown AE, Calandra T, Feld R, Pizzo PA, Rolston KV, Shenep JL, Young LS (2002). 2002 Guidelines for the use of antimicrobial agents in neutropenic patients with cancer. Clin Infect Dis.

[7] Ascioglu S, Rex JH, Pauw B, Bennett JE, Bille J, Crokaert F, Denning DW, Donnelly JP, Edwards JE, Erjavec Z, Fiere D, Lortholary O, Maertens J, Meis JF, Patterson TF, Ritter J, Selleslag D, Shah PM, Stevens DA, Walsh TJ (2002). Invasive Fungal Infections Cooperative Group of the European Organization for Research and Treatment of Cancer; Mycoses Study Group of the National Institute of Allergy and Infectious Diseases: Defining opportunistic invasive fungal infections in immunocompromised patients with cancer and hematopoietic stem cell transplants: An international consensus. Clin Infect Dis.

[8] Talcott JA, Siegel RD, Finberg R, Goldman L (1992). Risk assessment in cancer patients with fever and neutropenia: A prospective, two-center validation of a prediction rule. J Clin Oncol.

[9] Klastersky J, Paesmans M, Rubenstein EB, Boyer M, Elting L, Feld R, Gallagher J, Herrstedt J, Rapoport B, Rolston K, Talcott J (2000). The Multinational Association for Supportive Care in Cancer risk index: A multinational scoring system for identfying low risk febrile neutropenic cancer patients. J Clin Oncol.

[10] Guiguet M, Blot F, Escudier B, Antoun S, Leclercq B, Nitenberg G (1998). Severity of illness scores for neutropenic cancer patients in an intensive care unit: Which is the best predictor? Do multiple assessment times improve the predictive value?. Crit Care Med.

[11] Paganini HR, Aguirre C, Puppa G, Garbini C, Javier RG, Ensinck G, Vrátnica C, Flynn L, Iacono M, Zubizarreta P (2007). Febrile Neutropenia Study Group: A prospective, multicentric scoring system to predict mortality in febrile neutropenic children with cancer. Cancer.

[12] Pizzo PA, Ladisch S, Robichaud K (1980). Treatment of gram positive septicemia in cancer patients. Cancer.

[13] Raad II, Bodey GP (1992). Infectious complications of indwelling vascular catheters. Clin Infect Dis.

[14] Serra P, Santini C, Venditti M, Mandelli F, Martino P (1985). Superinfections during antimicrobial treatment with betalactam-aminoglycoside combinations in neutropenic patients with hematologic malignancies. Infection.

[15] Feld R, Goodman PJ, Higgins B, De Pauw BE, Deresinski S, Donnelly JP (1992). Prognostic factors fort he development of superinfection in febrile neutropenic cancer patients (abstract no 1695). Program and abstracts of the 32th Interscience Conference on Antimicrobial Agents and Chemotherapy.

[16] Mühlemann K, Wenger C, Zenhäusern R, Täuber MG (2005). Risk factors for invasive aspergillosis in neutropenic patients with hematologic malignancies. Leukemia.

